# OxDNA.org: a public webserver for coarse-grained simulations of DNA and RNA nanostructures

**DOI:** 10.1093/nar/gkab324

**Published:** 2021-05-01

**Authors:** Erik Poppleton, Roger Romero, Aatmik Mallya, Lorenzo Rovigatti, Petr Šulc

**Affiliations:** School of Molecular Sciences and Center for Molecular Design and Biomimetics, The Biodesign Institute, Arizona State University, 1001 South McAllister Avenue, Tempe, AZ 85281, USA; School of Molecular Sciences and Center for Molecular Design and Biomimetics, The Biodesign Institute, Arizona State University, 1001 South McAllister Avenue, Tempe, AZ 85281, USA; School of Molecular Sciences and Center for Molecular Design and Biomimetics, The Biodesign Institute, Arizona State University, 1001 South McAllister Avenue, Tempe, AZ 85281, USA; Department of Physics, Sapienza Università di Roma, P.le A. Moro 2 00185, Rome, Italy; School of Molecular Sciences and Center for Molecular Design and Biomimetics, The Biodesign Institute, Arizona State University, 1001 South McAllister Avenue, Tempe, AZ 85281, USA

## Abstract

OxDNA and oxRNA are popular coarse-grained models used by the DNA/RNA nanotechnology community to prototype, analyze and rationalize designed DNA and RNA nanostructures. Here, we present oxDNA.org, a graphical web interface for running, visualizing and analyzing oxDNA and oxRNA molecular dynamics simulations on a GPU-enabled high performance computing server. OxDNA.org automatically generates simulation files, including a multi-step relaxation protocol for structures exported in non-physical states from DNA/RNA design tools. Once the simulation is complete, oxDNA.org provides an interactive visualization and analysis interface using the browser-based visualizer oxView to facilitate the understanding of simulation results for a user’s specific structure. This online tool significantly lowers the entry barrier of integrating simulations in the nanostructure design pipeline for users who are not experts in the technical aspects of molecular simulation. The webserver is freely available at oxdna.org.

## Graphical Abstract

**Graphical Abstract:**
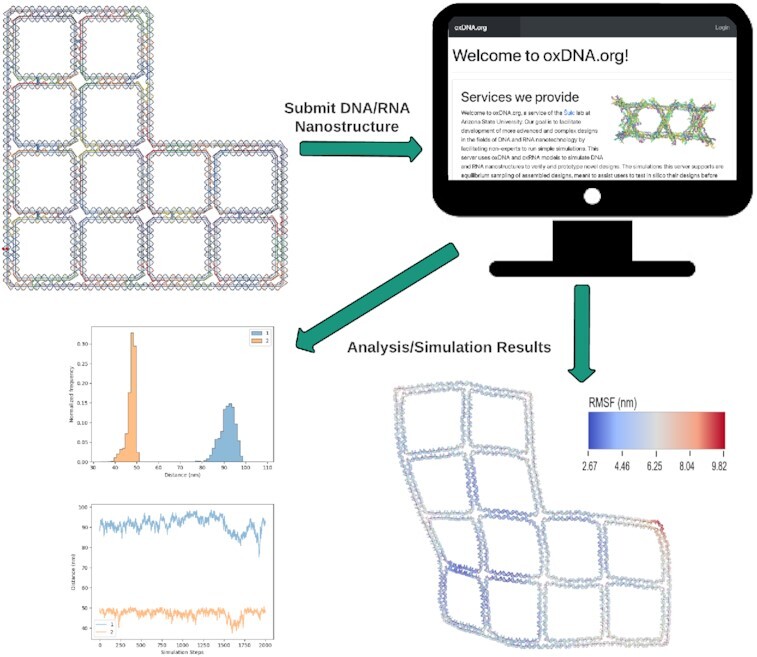
OxDNA.org is a server for DNA/RNA nanotechnology simulations. Nanostructures can be imported from popular nanotechnology design tools. The server then runs oxDNA/oxRNA simulations and provides a graphical user interface to visualize and analyze the results.

## INTRODUCTION

The field of nucleic acids nanotechnology uses DNA and RNA molecules as basic building blocks to construct nanoscale structures and devices. DNA and RNA have been chosen due to their programmability, which exploits the complementarity between corresponding bases (A–U/T, C–G) to design target nanostructures as overall free-energy minima of systems composed of self-assembling single DNA or RNA strands. Over the past four decades since its inception (1), the field has lead to the production of increasingly larger and more complex self-assembled structures with applications that include synthetic biology (2), nanopatterning (3), nanophotonics, drug delivery (4), diagnostics (5), immunotherapy (6) and vaccine development (7). Experimental techniques, such as fluorescent labeling, AFM and cryoEM are typically used to characterize the structures. However, the resolution of the experiments is limited, and most structures are typically designed empirically through a trial-and-error procedure, until the desired shape or structure property is achieved, which is time-consuming and costly.

**Figure 1.**
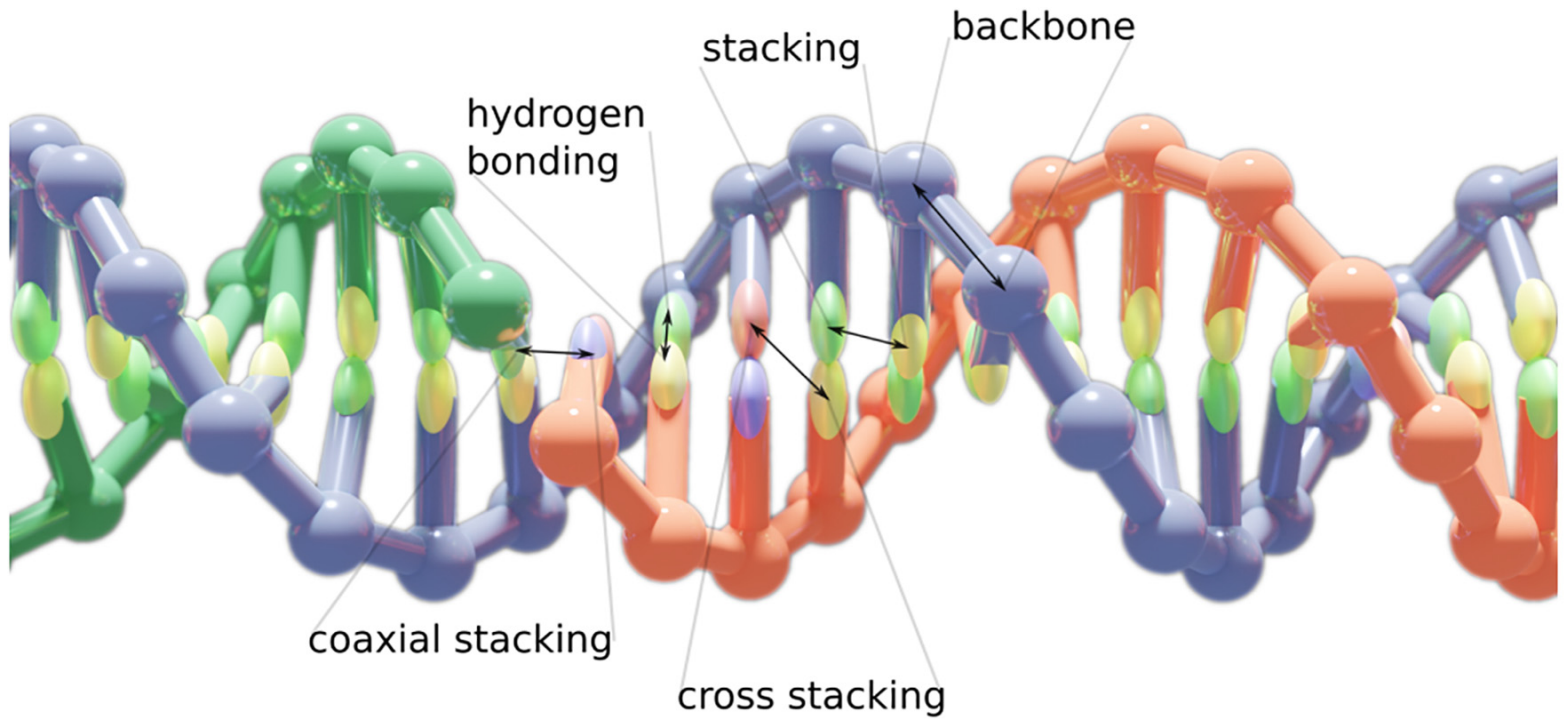
The oxDNA model is a coarse-grained one-bead-per-nucleotide model of DNA with empirically derived potentials between the beads which approximate the physical, mechanical and thermodynamic properties of double- and single-stranded DNA molecules. The electrostatic repulsion between nucleotides is implemented using a Debye-Hückel potential. The oxRNA model uses similar nucleotide-level representation for RNA molecules as oxDNA does for DNA.

An alternative is provided by computer simulations, which can offer detailed insight into the function and properties of DNA and RNA nanostructures. Using atomistic resolution models faces challenges due to the size of the systems (ranging from hundreds to tens of thousands of nucleotides), and to the long timescales involved in assembly as well as equilibrium states sampling for typical nanostructure designs. Hence, coarse-grained models, which use simplified representations that group multiple atoms into a single particle with effective parametrized interactions, have become increasingly popular to study nanostructures (8,9). Finite element-based computational studies of mechanical and structural properties of nanostructures (10,11) have also been developed. Among the most popular tools in the past few years have been the oxDNA and oxRNA models for DNA and RNA nanotechnology modeling (12–15). They represent each nucleotide as a single rigid body with empirically designed interactions that are parameterized to approximate basic structural, mechanical and thermodynamic properties of both single-stranded and double-stranded DNA/RNA (Figure 1). Where available, the models have been found to be in good agreement with experimental data and have been used in over 130 articles in the past ten years. Applications range from studies of biophysical properties of DNA and RNA to studies and rationalization of the function of DNA nanodevices, probing nanostructure design and simulations of their assembly (9,16–26). We note that the model does not currently support sequence-dependent structural properties of DNA such as AT-tract bending and sequence-specific stiffness. There are other approaches and models available which are specifically aimed at prediction of sequence-dependent effects in dsDNA structure (27–29).

There is, however, a steep learning curve in using the simulation code. It requires access to GPU-equipped servers, knowledge of command line environment and practical experience in setting up and evaluating molecular dynamics (MD) simulations, which typically requires at least basic programming expertise as well. One of the most common use cases of oxDNA and oxRNA is to prototype and test novel nanostructure designs in equilibrium sampling simulation. To ease the use of the tool and also make it accessible to a broader user base, we introduce here a public webserver, oxDNA.org, with a GUI that integrates automated simulation setup and subsequent evaluation of the resulting trajectory in an intuitive user-friendly environment. We provide here the description of the simulation setup and analysis workflow, as well as information about the models and the file formats used. Further tutorials and examples of how to use the server are provided online. Similar to the CanDo webserver (10), our server allows upload of a nanostructure in a specified file format. As opposed to CanDo, which uses finite-element methods to assess structure flexibility and predict mean structure, oxDNA represents the strands on nucleotide-level, thus also allowing for breaking or formation of base pairs during the simulation and more accurate representation of single-stranded regions. We also support a larger set of analysis tools that also includes distance and angle distribution, and the server also supports RNA simulations via the oxRNA model. However, since the model uses molecular dynamics to sample the structures, it takes longer to evaluate the nanostructures than CanDo.

## MATERIALS AND METHODS

### Server data processing

**Figure 2.**
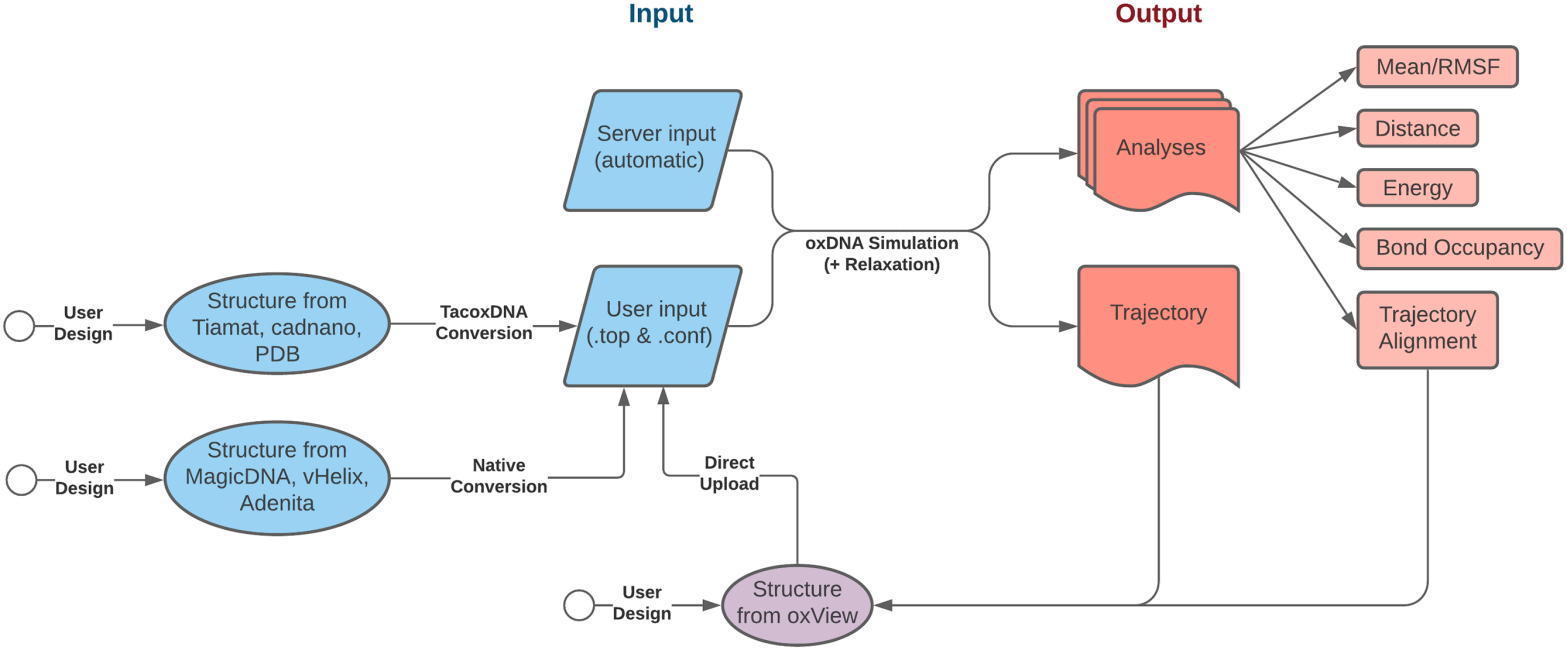
Workflow of the oxDNA.org pipeline.

OxDNA.org runs the public release of the oxDNA simulation code that implements the oxDNA2 and oxRNA models. It facilitates user interaction via a graphical user interface (GUI) that automatically generates parameters for the simulation and simplifies post-processing. The data workflow used by oxDNA.org is shown in Figure 2. The details about the parametrization of the models and the computational implementation can be found in previous work (12–15,30). The oxDNA.org webserver brings together, for the first time, structure relaxation, simulation, visualization and post-processing in a single GUI environment. Users provide the DNA and RNA structure input files in the oxDNA format and are presented with the option to choose a limited number of simulation parameters:

- Input files – An oxDNA configuration and topology file pair that define the structure that will be simulated
- Job Name – The name you would like to give the job
- Interaction Type – Whether to use the oxDNA2 or oxRNA force field when running the simulation
- Salt Concentration – The monovalent ion concentration in molar. OxDNA uses the Debye-Hückel electrostatic model to implicitly model reduction in backbone electrostatic repulsion due to the presence of monovalent ions. Magnesium interacts with DNA and RNA in a non-uniform, site-specific manner and therefore is not included in the oxDNA model. Previous studies (31) have shown that high monovalent ion concentration in the simulation produce results very similar to those found in experiments containing standard levels of magnesium ions.
- Steps – The number of steps to run the simulation (running time = steps · timestep).
- Temperature – The temperature of the simulation (set with an Andersen-like thermostat).
- Relaxation – If checked a relaxation protocol will be run prior to the production simulation. The protocol comprises a Monte-Carlo (MC) relaxation and an MD relaxation.

The following additional parameters are available for the relaxation protocol:

- MC Steps – The number of steps to run the MC relaxation for. This is mostly used to relax overlapping particles and stretched bonds. If there are not many of these in the input structure, this can be shorter than the default.
- MD Steps – The number of steps to run the MD relaxation for. If structures do not completely relax, this step should be made longer.
- MD Timestep – The timestep of the MD relaxation. If structures fail to relax or explode during the relaxation, it can be helpful to lower this value.

The following parameters are also available in the ‘advanced parameters’ section for the production simulation:

- Backend – Run using the CPU or CUDA (GPU) backend. For structures over a couple hundred nucleotides the CUDA backend will be significantly faster. The server is currently equipped with 8 GPUs and 20 CPU cores, so small structures should be run on CPU to leave GPU capacity for larger structures.
- Simulation Timestep – The timestep of integration for the MD simulation. This number should not be set higher than 0.003 to avoid numerical instability.
- External Force File – The user may upload an external force file to add additional forces. All valid oxDNA force files are supported, the full list of formats can be found in the oxDNA documentation (dna.physics.ox.ac.uk). Mutual traps, the most commonly used type of force to pull two separated strands into proximity, can be generated interactively by oxView (32) by selecting which nucleotides should be paired together. Alternatively, the force files can also be used to pin a specific nucleotide to a given position, or to introduce a 2D plane in the simulation box, as described in the oxDNA documentation.
- Average Sequence Model – By default, oxDNA and oxRNA use the same (averaged) sequence strength for A–T(U) and C–G base pairs and for stacking interactions. Switching this option will run the model with sequence-dependent strengths for A–T and G–C bonds (or A–U, G–C and G–U for RNA) as well as with sequence-dependent stacking interaction strengths.
- Mismatch Repulsion – The oxRNA force field is known to over-stabilize mismatches between paired segments. This introduces an additional repulsion force between non-complementary bases to reduce such incidents.
- Print Conf Interval – The frequency with which the simulation will print its current configuration to the trajectory file. The default number was chosen to obtain configurations that are, on average, uncorrelated. OxDNA limits data output to 1MB per second, so the maximum print rate depends on the size of the structure.
- Print Energy Interval – The frequency with which the simulation will print its current energy to the energy file.

The default parameters were chosen to provide a good balance between runtime and sampling and in most cases will result in a trajectory with uncorrelated energies between subsequent configurations. The default parameters will run a DNA simulation at 20°C and 1 M sodium concentration for 10^9^ MD steps with a timestep of integration of 0.001 simulation time units. Note that, due to the inherent nonlinearity in coarse-graining, an exact time correspondence with experiments cannot be established (23). Based on direct unit conversion the default running time corresponds to 3.03 μs. Previously, the correspondence between the simulation time and experimental time was roughly based on the diffusion of a 14-mer (33,34), which is about 100 times faster in simulation than what has been measured experimentally. Another possible correspondence between the simulation and the experimental times was obtained by comparing the DNA duplex hybridization rate (23), yielding a factor of ∼3000. Hence, the 10^9^ steps can correspond up to roughly 10 ms in real time. However, different processes might scale with different ratios and this number should be only considered as a very crude estimate.

Relaxation will be required for most structures exported from DNA/RNA nanostructure design software prior to starting the oxDNA simulation, since most design software presents an idealized version of DNA structures that is not strictly accurate from a physical perspective. For example, the respective parts of the structures might be drawn on a lattice and the nucleotide positions in the design interface might violate the length constraints imposed by covalent bonds. Another common problem with structures directly exported from design tools is that some nucleotides are positioned too close to each other, leading to steric clashes. An MD simulation cannot be directly started from such configurations, as the large forces due to the unphysical conformations would lead to numerical instabilities. OxDNA.org hence implements the relaxation scheme described in (35) where a short Monte-Carlo simulation is first performed to reduce excluded-volume clashes and shorten stretched bonds. The structure is then relaxed using a longer MD simulation with a highly-coupled Bussi-Donadio-Parrinello thermostat (36) and a modified backbone potential which reduces the possibility of numerical instabilities. The default relaxation parameters on the webserver are very generous in order to facilitate most user submissions. Structures that are already in a near-physical state can be run faster by reducing the number of steps in the relaxation, while some structures that are very far from their expected configuration may require more aggressive methods beyond the scope of oxDNA.org such as rigid-body dynamics (32) which can be performed using oxView, mrDNA (8), or interactive relaxation using ox-serve (37).

Simulations of a full-sized DNA origami (∼10 000 nucleotides) take about 3 days to run on oxDNA.org with the default parameters, with runtime scaling approximately linearly with the total number of nucleotides. Users are allowed to submit up to four simulations at a time, and the trajectories are kept on the server for one week after completion. The trajectories are stored in a compressed 7zip format, however for a origami-sized structure the files will still be ∼3GB, so ensure sufficient time to download the results. The server is currently equipped with 8 NVIDIA RTX 2080 Ti GPU cards with plans to expand capacity in the future.

### Software

The web frontend/backend uses a Flask (Python3)/Angular 1.8 (JavaScript)/Bootstrap(CSS+HTML) stack. The main code of oxDNA is written in C++ and CUDA and can be downloaded from (dna.physics.ox.ac.uk). OxView (sulcgroup.github.io/oxDNA-viewer/) is a single-page Three.js (JavaScript) application used for visualization and editing of oxDNA structures. Analysis is performed using the Python-based oxDNA_analysis_tools package (github.com/sulcgroup/oxDNA_analysis_tools). Examples of outputs are demonstrated in the following section.

## RESULTS AND DISCUSSION

### Input files for the server

**Figure 3.**
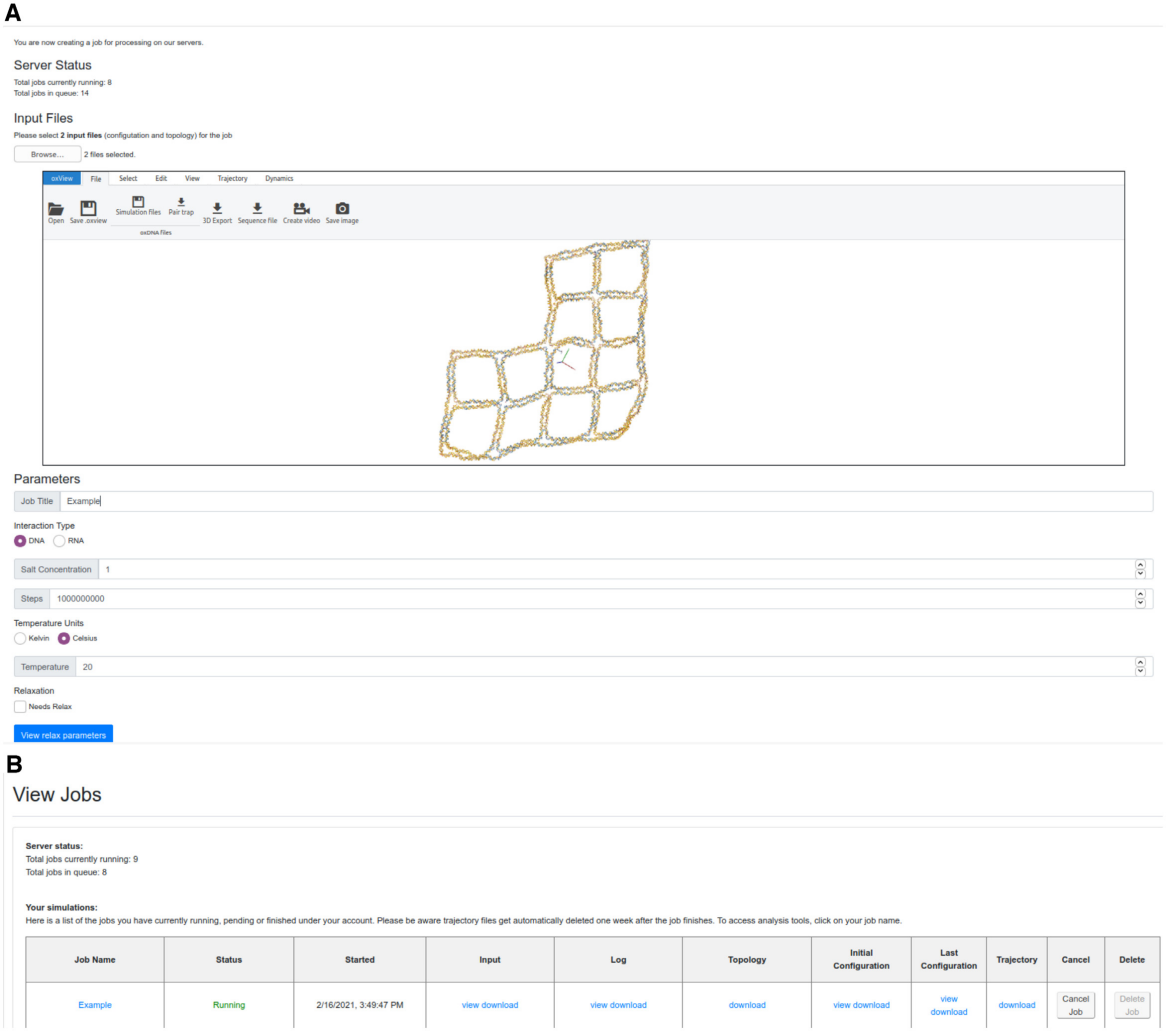
Submitting a job on oxDNA.org The Job submission interface showing the uploaded structure in oxView and the parameter setting (**A**). The job status page where users can check the status of their jobs as well as download the original input files as well as the output files and logs. Clicking on the job name will take the user to the analysis page for that job (**B**).

The job submission form on oxDNA.org (Figure 3) requires two files: configuration and topology. Both files are described in detail in the oxDNA documentation on dna.physics.ox.ac.uk. Briefly, the configuration file header has three lines that contain, respectively, the timestep, simulation box size, and total, potential and kinetic energy per particle for the given configuration. Then, the file contains one line per nucleotide. Each line contains the nucleotide position, the normal particle orientation vectors $\mathbf {a}_1$ and $\mathbf {a}_3$ of the reference frame of the nucleotide, and the velocity and angular velocity vectors. The topology file contains connectivity information defining which particles are connected together to form strands as well as the nucleoside identity of each particle. The first line of the topology file shows the total number of nucleotides and strands. Then, for each nucleotide, its corresponding line in the topology file lists the strand id that the nucleotide is part of, the base identity (A,C,G, T or U for RNA), the id of the nucleotide’s 3′ neighbor along the strand and then the id of the nucletotide’s 5′ neighbor. The nucleotides in the topology file are listed 3′ to 5′ (note the backwards convention) for each respective strands. If a nucleotide does not have a 3′ or 5′ neighbor, the id of the neighbor is listed as –1. The ids of the strands start from 1, the ids of the nucleotides start from 0.

There are a variety of popular design tools in the DNA/RNA nanotechnology field (38–42) that can be used to create a starting configuration for an oxDNA simulation. Some (like Adenita, MagicDNA or vHelix) have built-in exporters to the oxDNA format while others (caDNAno, Tiamat, or PDB format) can be converted using TacoxDNA (tacoxDNA.sissa.it) (43). OxDNA files can be edited using the oxView tool, on the main website (sulcgroup.github.io/oxDNA-viewer/). Users may optionally include an external force file which defines an external potential that acts on certain particles in the simulation. Most commonly used are mutual traps, which add an external spring potential of a given stiffness and equilibrium length between two particles. These files can be generated using oxView or oxDNA_analysis_tools or can be manually written based on the template provided in the documentation.

When submitting a job, users can either sign up for an account to keep track of all their submissions in one place or submit anonymously, in which case the user should bookmark the job output link for later access. Users who create an account may opt-in to emails when jobs complete and will receive email reminders about stale files that will soon be removed from the server.

### Server output result

**Figure 4.**
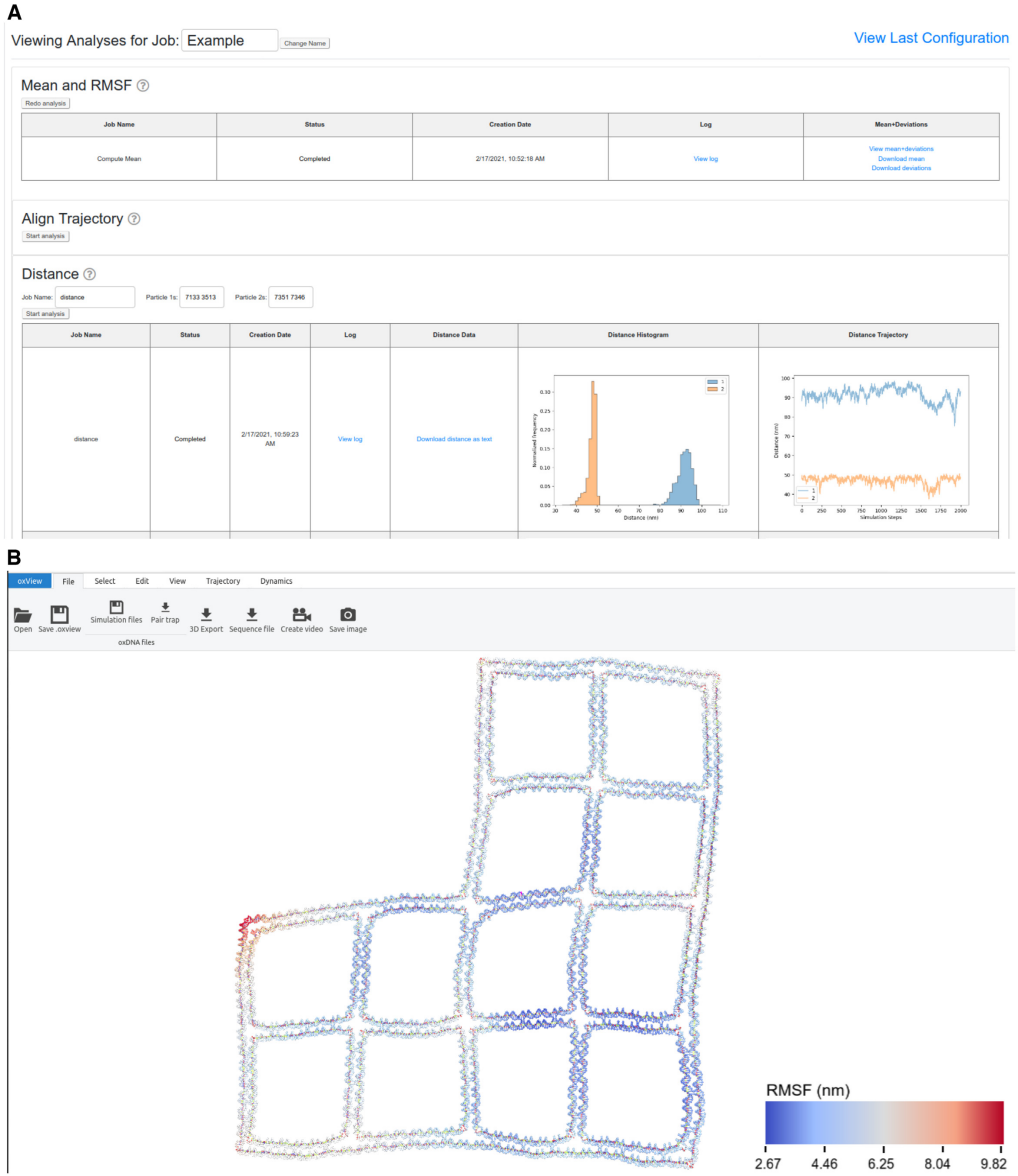
Analyzing a completed job The job analysis interface showing a few of the options available to users when their simulation finishes. (**A**) In this example, a wireframe DNA origami from (44) was simulated and, when finished, the mean structure with RMSF and the end-to-end distances of two of the edges were calculated. Clicking on the ‘view’ options will open the structure in a separate oxView tab allowing the user to interactively explore their results (**B**).

There are two pages of outputs from simulation jobs run on the server (Figure 4). The first is a summary table of all jobs run by the user which contains links to download the initial configuration and topology files submitted, as well as the simulation input file and job logs generated by the server. The user can further view or download the last configuration output by the simulation and download a zip archive containing the entire simulation trajectory. By clicking on the job name, the user will be taken to the analysis page where they will find many options for post-processing of their simulation trajectory. This page is a GUI implementation of many of the scripts found in oxDNA_analysis_tools. The following scripts are available through the GUI:

- Mean and RMSF – Uses single value decomposition (SVD) alignment to calculate the mean position of every nucleotide, then again analyzes the trajectory to get the root mean squared fluctuation (RMSF) of each particle from its mean position.
- Align Trajectory – Uses SVD to align all frames in the trajectory to the first. Produces a clearer view of fluctuations when viewed or converted to a movie using oxView. This creates a trajectory file about 2/3 the size of the original, so is recommended prior to downloading.
- Distance – Calculates the distances between two lists of particles (the first particle id in ‘particles 1’ is compared with the first particle id in ‘particles 2’ etc.) Results are provided as both a histogram and line plot format in addition to as a text file.
- Energy – Creates plots out of the simulation energy file showing the average potential energy per particle. Fluctuation around a constant value is the simplest way to determine whether or not a structure has been properly equilibrated.
- Bond Occupancy – Calculates the percentage of trajectory frames in which the bonds present in the first configuration are subsequently present. This is useful for prototyping structures, as improperly designed regions will be unstable and prone to breaking base pairs.
- Duplex Angles – This is split into two sections, the first calculates the orientation of all duplexes in the structure. The second takes as input the starting nucleotide ids of duplexes and uses the output file from the previous script to calculate the angle between duplexes that start with those particles. The input and output format is the same as the distance script.

‘Mean and RMSF’, ‘Energy Plotter’, and ‘Bond Occupancy’ give a good summary of how the structure behaves and what fluctuation modes the structure encountered during the simulation. Base pair occupancy in particular is something that rapid equilibrium structure prediction algorithms such as CanDo(10) and SNUPI(11) cannot predict. Distance and Angles serve to ask specific questions about how regions of the structure behave relative to one another, allowing the user to accurately discern parameters such as ideal locations for FRET pairs or explore 3D curvature that is not visible on AFM images. All results from the server can be downloaded by clicking the download link.

The webserver has been tested on all major browsers and operating systems (Table 1). The original oxDNA code is supported only for Unix-based systems, so this improves accessibility to those using Windows environments.

**Table 1.** Browser Compatibility: oxDNA.org works in all major browsers and operating systems

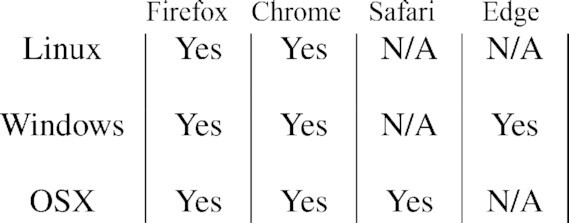

## CONCLUSION

OxDNA.org simplifies the oxDNA/oxRNA simulation pipeline and makes a tool previously limited to Unix command line execution usable to non-expert users via a GUI. OxDNA has long been a popular tool for the prototyping of DNA and RNA nanotechnology structures, and we hope that the simplification of the interface and the implementation of automatic relaxation will allow this tool to find wider adoption among traditionally experiment-only groups and that simulation-probing will become a standard step within the nucleic acid nanostructure design and characterization process. The webserver implementation and scripts are also freely available under GNU Public License at github.com/sulcgroup/oxdna-web. The webserver code is available for anyone to setup their own lab server for oxDNA simulations, as well as setup mirror sites of oxDNA.org to increase the resource availability to the community. The package is optimized for nginx webserver on Linux operating system, and the installation and setup details are provided on the github page.
